# Supplementary material for: Dual-task walking reduces lower limb range of motion in individuals with Parkinson’s disease and freezing of gait: But does it happen during what events through the gait cycle?
Source: PLoS One. 2020 Dec 8;15(12):e0243133. doi: 10.1371/journal.pone.0243133 (PMC7723257; doi:10.1371/journal.pone.0243133)
Supplement: Attachment — Submitted filename: Response to Reviewers.docx [file pone.0243133.s003.docx]

**Response to Reviewers**

*PONE-D-20-03253R1*

*Dual-task walking reduces lower limb range of motion in individuals with Parkinson’s Disease and Freezing of Gait. But does it happen during what events through the gait cycle?*

**Review Comments to the Author:**

Reviewer #1: This study investigates sagittal-plane joint range of motion (RoM) and kinematics during dual-task gait in individuals with Parkinson’s disease (PD) that have freezing of gait. Although the revised manuscript is overall responsive to the previous critiques, one major concern still remains: how much of the identified differences in RoM and kinematics are simply a result of walking at a slower speed?

R.: We’d thank the reviewer’s contributions to our study. This point is quite relevant. Actually, anyone walking in dual-task will face some degree of gait speed reduction. In order to answer this question in a more precise way, we performed a multiple linear regression analysis. With this analysis, we investigated the contribution of gait speed on the range of motion in each condition separately (*Cohen & Cohen, 1983)*. Our results showed that gait speed was able to explain 70.8% and 49.9% of the hips RoM during single and dual-task gait, respectively, 68.2% and 61.2% of the knees, and 28% and 36.8% of ankles. These results show that gait speed has an important influence on RoM, mainly in hips and knees. We made several modifications across the paper to include and discuss this point. Please see methods (page 7), results and table 3 (pages 9 and 11).

*Ref.: Cohen, J. & Cohen, P. (1983). Applied multiple regression/correlation analysis for the behavioral sciences (2nd ed.). Hillsdale, NJ: Lawrence Erlbaum Associates. [View](http://www.amazon.com/gp/product/0898592682?ie=UTF8&tag=statissoluti-20&linkCode=as2&camp=1789&creative=9325&creativeASIN=0898592682)*

A significant portion of the Discussion section is focused on the mechanical implications of individual joint kinematic differences. However, there is no discussion of how these differences may simply be due to walking at a slower speed. The subject cohort walked significantly slower in the dual-task condition. The authors also identified some differences in the dual task compared to single task conditions in both joint RoM and kinematics. The reduction in walking speed was significantly correlated with reduced RoM in both conditions. It may be that these individuals reduced their walking speed (e.g., to increase stability?) and as a result their kinematics differed. If this is true, statements such as “lack of ankle plantarflexion would be the major problem related to gait impairments in the swing periods of dual-task gait” would not be true. To tease out how much of these kinematic differences are speed related would require individuals to walk at matched speeds between conditions. The Discussion section should be revised in light of this important point.

R.: We agree with the reviewer’s comment. In fact, gait speed may have an influence on RoM when individuals change their attention to another task (i.e. dual-task) and seek for stability. In order to attend this relevant discussion, we performed a regression analysis and included this analysis through the manuscript as detailed above. Moreover, we modified our discussion based on this new idea. The statement that the reviewer mentioned was excluded (page 15) and new points were added regarding the gait speed contribution on lower limb RoM, as well new references about this topic. Please see detailed below:

*“Moreover, previous studies discuss an important influence by gait speed on lower limb kinematics [36], concluding that gait speed is angle dependant and dependant on the phase of the gait cycle [37]. It means that the RoM of lower limb joints increase with a faster walk [36]. In order to assess the contribution of gait speed on lower limb RoM in our results we performed a regression analysis, although the best way to inform the speed contribution would be to require individuals to walk at matched speeds between conditions. It seems that a slower or faster walking speed has a strong relation on how much individuals increase the RoM of lower limbs, especially for hips and knees. We hypothesize that when individual focus on another activity (dual-task gait), they seem to reduce the gait speed in order to increase stability and as a result the RoM of lower limb joints decrease.”*

*Ref.:*

*36. Han Y, Wang X (2011) The biomechanical study of lower limb during human walking. Science China Technological Sciences 54: 983-991.*

*37. Hanlon M, Anderson R (2006) Prediction methods to account for the effect of gait speed on lower limb angular kinematics. Gait Posture 24: 280-287.*

**Minor Comments:**

Line 80-81: The authors state that a previous study examined RoM in people with PD without FOG - what did they find?

R.: We added the results about the study mentioned. Please see the modifications bellow:

*“A previous study evaluated lower limb RoM (sagittal plane) on people with PD without FOG and control subjects during a single gait [16]. Individuals with PD showed a reduced RoM of hips, knees, and ankles (3 to 5 degrees less, approximately) during a walking performance when compared to control subjects [16].”*

Line 85-86: The authors state that using discrete parameter statistics it is impossible to visualize movement trajectories into the specific gait phase. “Impossible” is too strong of a statement, as this could be carefully done by analyzing average or change in kinematics within specific sub-phases of gait.

R.: We agree with the reviewer statement. We rewrite this sentence as follows:

*“Traditional gait analysis generally quantifies lower limb RoM as the average of the entire gait cycle through discrete parameter statistics, which is challenging to visualize movement trajectories into specific gait phases [**2**,**14**,**17**].”*

Line 90: Missing a word - “pattern and location of joint kinematic during a whole movement cycle”

R.: Done as suggested.

*“SPM is a method able to analyze one-dimensional (1D) data and identify the exact pattern and location of joint kinematics during the entire gait cycle.”*

Line 331: replace “exact gait events” with “exact gait phases”

R.: Done as suggested.
