# Supplementary material for: Dual-task walking reduces lower limb range of motion in individuals with Parkinson’s disease and freezing of gait: But does it happen during what events through the gait cycle?
Source: PLoS One. 2020 Dec 8;15(12):e0243133. doi: 10.1371/journal.pone.0243133 (PMC7723257; doi:10.1371/journal.pone.0243133)
Supplement: Attachment — Submitted filename: Response to Reviewers.docx [file pone.0243133.s004.docx]

**Response to Reviewers**

*PONE-D-20-03253R2*

*Dual-task walking reduces lower limb range of motion in individuals with Parkinson’s Disease and Freezing of Gait. But does it happen during what events through the gait cycle?*

**Additional Editor Comments (if provided):**

Dear authors,

Thank you for taking the time to respond to the reviewer's critiques. While they have been generally satisfied, there are two changes that are required to meet the criteria for publication.

First, I encourage you to please more fully address the reviewer's concern regarding the significant limitation in the interpretation of these results due to confounding between the independent variable of task (dual vs. single) and gait speed (slower vs. faster). The inclusion of the regression modeling provides insight to this relationship, but in the context of this experimental design, where task was controlled but gait speed was not, the potential remains for confounding between these two interacting variables. Therefore, please add a clear paragraph to the discussion similar to the following.

"One important limitation is that although we demonstrated changes in lower limb kinematics during dual-task gait when compared to single gait, particularly during toe off and heel strike, reductions in gait speed were also observed in the dual-task condition, which raises the potential for confounding. It is unknown whether these changes in lower limb kinematics result directly and uniquely from the dual-task condition, or whether similar changes would be observed in a single-task condition with slower gait speed."

Additionally, in multiple places the authors refer to the study as "a cross sectional study." Because the primary independent variable is task condition within a subject (single dual-task vs. dual-task), rather than some other factor across subjects (say, FOG vs. NO FOG), it would be more precise to refer to the study as an "observational within-subjects study." Please change this language in the Abstract, Ethics Statement, and Methods.

R.: We would like to thank the editor for his contributions to our manuscript. Indeed, to find out how much lower limb kinematics are speed-related, individuals should walk at matched speeds in dual and single-task conditions. The gait speed was not controlled in our study and it might be a potential for confounding. We highlighted this limitation in the discussion section (pages 16-17). We apologize for missing this important critique. Moreover, we changed the study design to "observational within-subjects study" in the Abstract, Ethics Statement, and Methods.
